# Cholesteatoma labyrinthine fistula: prevalence and impact^☆^

**DOI:** 10.1016/j.bjorl.2018.01.005

**Published:** 2018-03-09

**Authors:** Letícia P. Schmidt Rosito, Inesângela Canali, Adriane Teixeira, Mauricio Noschang Silva, Fábio Selaimen, Sady Selaimen da Costa

**Affiliations:** a Universidade Federal do Rio Grande do Sul (UFRGS), Hospital de Clínicas, Departamento de Otorrinolaringologia, Cirurgia de Cabeça e Pescoço, Porto Alegre, RS, Brazil; b Universidade Federal do Rio Grande do Sul (UFRGS), Hospital de Clínicas, Departamento de Oftalmologia e Otorrinolaringologia, Porto Alegre, RS, Brazil

**Keywords:** Labyrinthine fistula, Cholesteatoma, Hearing loss, Fístula labiríntica, Colesteatoma, Perda auditiva

## Abstract

**Introduction:**

Labyrinthine fistula is one of the most common complications associated with cholesteatoma. It represents an erosive loss of the endochondral bone overlying the labyrinth. Reasons for cholesteatoma-induced labyrinthine fistula are still poorly understood.

**Objective:**

Evaluate patients with cholesteatoma, in order to identify possible risk factors or clinical findings associated with labyrinthine fistula. Secondary objectives were to determine the prevalence of labyrinthine fistula in the study cohort, to analyze the role of computed tomography and to describe the hearing results after surgery.

**Methods:**

This retrospective cohort study included patients with an acquired middle ear cholesteatoma in at least one ear with no prior surgery, who underwent audiometry and tomographic examination of the ears or surgery at our institution. Hearing results after surgery were analyzed according to the labyrinthine fistula classification and the employed technique.

**Results:**

We analyzed a total of 333 patients, of which 9 (2.7%) had labyrinthine fistula in the lateral semicircular canal. In 8 patients, the fistula was first identified on image studies and confirmed at surgery. In patients with posterior epitympanic and two-route cholesteatomas, the prevalence was 5.0%; and in cases with remaining cholesteatoma growth patterns, the prevalence was 0.6% (*p* = 0.16). In addition, the prevalence ratio for labyrinthine fistula between patients with and without vertigo was 2.1. Of patients without sensorineural hearing loss before surgery, 80.0% remained with the same bone conduction thresholds, whereas 20.0% progressed to profound hearing loss. Of patients with sensorineural hearing loss before surgery, 33.33% remained with the same hearing impairment, whereas 33.33% showed improvement of the bone conduction thresholds’ Pure Tone Average.

**Conclusion:**

Labyrinthine fistula must be ruled out prior to ear surgery, particularly in cases of posterior epitympanic or two-route cholesteatoma. Computed tomography is a good diagnostic modality for lateral semicircular canal fistula. Sensorineural hearing loss can occur post-surgically, even in previously unaffected patients despite the technique employed.

## Resumo

**Introdução:**

A fístula labiríntica é uma das complicações mais comuns associadas ao colesteatoma. Representa uma perda erosiva do osso endocondral que recobre o labirinto. As razões para a ocorrência da fístula labiríntica induzida pelo colesteatoma ainda são mal compreendidas.

**Objetivo:**

Avaliar pacientes com colesteatoma, a fim de identificar possíveis fatores de risco ou achados clínicos associados à fístula labiríntica. Os objetivos secundários foram determinar a prevalência de fístula labiríntica no estudo de coorte, analisar o papel da tomografia computadorizada e descrever os resultados auditivos após a cirurgia.

**Método:**

Este foi um estudo de coorte retrospectivo. Foram incluídos pacientes com colesteatoma adquirido de orelha média em pelo menos um lado sem cirurgia prévia que haviam sido submetidos à audiometria e tomografia computadorizada de orelha ou cirurgia em nossa instituição. Os resultados auditivos após a cirurgia foram analisados de acordo com a classificação de fístula labiríntica e da técnica empregada.

**Resultados:**

Analisamos um total de 333 pacientes, dos quais 9 (2,7%) apresentavam fístula labiríntica no canal semicircular lateral. Em 8 pacientes, a fístula foi identificada na tomografia computadorizada e confirmada durante a cirurgia. Em pacientes com colesteatomas epitimpânicos posteriores e de via dupla, a prevalência foi de 5,0%; e nos casos com padrão de crescimento de colesteatoma remanescente, a prevalência foi de 0,6% (*p* = 0,16). Além disso, a taxa de prevalência de fístula labiríntica entre pacientes com e sem vertigem foi de 2,1. Dos pacientes sem perda auditiva neurossensorial antes da cirurgia, 80,0% permaneceram com os mesmos limiares de condução óssea, enquanto 20,0% progrediram para perda auditiva profunda. Dos pacientes com perda auditiva neurossensorial antes da cirurgia, 33,33% permaneceram com a mesma deficiência auditiva, enquanto 33,33% apresentaram melhora da média de dos limiares de condução óssea aos tons puros.

**Conclusão:**

A fístula labiríntica deve ser descartada antes do procedimento cirúrgico, particularmente nos casos de colesteatomas epitimpânicos posteriores e de dupla via. A tomografia computadorizada é uma boa modalidade diagnóstica para a fístula do canal semicircular lateral. A perda auditiva neurossensorial pode ocorrer pós-cirurgicamente, mesmo em pacientes previamente não afetados, a despeito da técnica empregada.

## Introduction

Acquired middle ear cholesteatoma is a gradually expanding destructive epithelial lesion of the temporal bone, resulting in progressive erosion of adjacent bony structures.^1^ Labyrinthine fistula (LF) is one of the most common complications associated with cholesteatoma.^2^ It represents an erosive loss of the endochondral bone overlying the labyrinth. The loss of the overlying protective bone allows pressure or mass-induced motion of the underlying endosteum, perilymph, and by contiguity, the endolymphatic compartment, evoking vestibular and sometimes auditory symptoms.^1^

The etiology of cholesteatoma-induced LF in some patients is still poorly understood. Cholesteatoma growth pattern, age of the patient, and time of the disease, are possible factors that influence the site of bone erosion and aggressiveness of the disease, which may lead to development of LF.

Pre-operative detection of LF is of great importance to ear surgeons. LF may not be associated with any specific symptom or sensorineural hearing loss (SHL) before surgery,^3^, ^4^, ^5^, ^6^ nevertheless, proper management is essential to prevent poor outcome. The applicability of CT scan in pre-operative LF evaluation remains controversial. Study results differ based on different CT methods and selection criteria of patients.^7^, ^8^

Our main objective was to evaluate patients with acquired middle ear cholesteatoma without previous treatment, in order to identify possible risk factors or clinical findings associated with LF. Secondary objectives were to determine the prevalence of LF in the study cohort, to analyze the role of high resolution computed tomography (CT) in predicting LF in this population and to describe the hearing results after surgery.

## Methods

Patients diagnosed with acquired middle ear cholesteatoma at a tertiary hospital between August 2000 and March 2016 were included in the study.

The inclusion criterion was presence of acquired cholesteatoma in at least one middle ear. Exclusion criteria included history of any previous ear surgery except tympanostomy for ventilation tube placement and inability to undergo cleaning and videotoscopy for appropriate imaging. The study population was divided into the pediatric group comprising patients aged 0 to 18 years, 11 months, and 30 days (United Nations Convention on the Rights of Children, 1989), and the adult group, with patients aged ≥ 19 years.

For every patient, a detailed clinical history was recorded. We questioned every one about the existence of vertigo complaints. Otological examinations were performed after carefully cleaning the ear canals. Fiberoptic otoendoscopy (0° and 4 mm otoendoscope, Karl Storz GmbH, Tuttlingen, Germany) was performed in both ears and images were recorded sequentially with Power Director version 7 (CyberLink Corporation, Taipei, Taiwan).

The recorded images were independently reviewed by the same researcher who was blinded to symptoms or other findings related to the fistulas. Cholesteatoma growth pattern was classified as follows: Posterior Epitympanic Cholesteatoma (PEC), confined to the pars flaccida; Posterior Mesotympanic Cholesteatoma (PMC), in the posterosuperior quadrant of the pars tensa; Anterior Epitympanic Cholesteatoma (AEC), originating cranially and anteriorly to the malleus head; two-route, involvement of both, the pars flaccida and the pars tensa; and undetermined, precise growth pattern unidentifiable by videotoscopy.

All patients underwent pure tone audiometry (AD 27 audiometer, Interacoustics AS, Assens, Denmark; TDH-39 supra-aural earphones, Telephonics Corporation, Farmingdale, NY, USA). For measuring bone conduction (BC) thresholds, a BC transmitter was placed on the mastoid bone. Narrow-band masking noise was applied when needed. In young children, in whom reliable measurements are difficult to obtain, play-conditioned audiometry with supra-aural earphones was performed. When necessary, pure tone audiometry was concluded after two sessions to confirm the results.

Air conduction (AC) thresholds were measured at the following frequencies: 250; 500; 1000; 2000; 3000; 4000; 6000; and 8000 Hz. BC thresholds were measured at the following: 500; 1000; 2000; 3000; and 4000 Hz. Further, air-bone gaps (ABGs) were calculated from the differences between the AC and the BC thresholds. The pure tone average (PTA) of the AC and BC thresholds and ABGs were calculated as the mean of the following: 500; 1000; and 2000 Hz. The audiometries were performed at the first evaluation and subsequently at six months to one year after the surgery. Differences bigger than 10 dB between BC thresholds’ PTA before and after surgery were considered improvement or impairment of the hearing loss, respectively.

As preparation for surgery, high resolution CT scan was conducted in every patient before the procedure.

In our department, most patients with LF are routinely submitted to open tympanomastoidectomy. When identified during the surgery, fistulas were classified according the degree of labyrinth involvement, as described in Table 1 (modified from Dornhoffer and Milewski).^9^ We only considered as LF the Stages II or III.

**Table 1:** Modified intraoperative classification of Dornhoffer and Milewski.

Type	Description
Type I	Perilymphatic membrane covered with bone; i.e., blue line only.
Type II	Perilymphatic membrane exposed.
Type III	Perilymphatic membrane eroded onto organs or cholesteatoma inside the labyrinth.

The classification between Type II or III was only possible during surgery. After finishing the mastoidectomy and completely cleaning the mastoid bowl, only a small area of matrix lying over the potential fistula was left behind. Attention was then directed to this area; working under higher magnification, the area was gently explored: if the matrix could be safely peeled off the membranous labyrinth, leaving it intact, the fistula was classified as Type II; If there was not a clear cleavage plan between the matrix and the membranous labyrinth (jeopardizing its integrity during dissection) or there was a deep indentation of the matrix inside the labyrinth, the fistula was classified as Type III. In these situations, exfoliated keratin and debris were cautiously sucked off and the matrix was left intact.

The surgical techniques used for management of LF were: (A) complete removal of the cholesteatoma matrix in Type II fistula or (B) partial retention of matrix in situ in Type III fistula. In cases of complete removal, once the fistula was identified, the matrix was sharply cut over the fistula followed by complete mastoid drilling; subsequently, the matrix was carefully removed without sucking. Reconstruction was immediately performed with temporal fascia and bone pate. All patients were administered intra- and post-operative corticosteroids and antibiotics.

Statistical analysis was performed by using SPSS software (SPSS, Chicago, IL). Qualitative variables were analyzed by Chi-square or Fisher's exact test. Quantitative variables were compared with the Mann–Whitney *U*-test or Wilcoxon test, when indicated. All tests were two-tailed, and the level of significance was set at *p* ≤ 0.05.

We also calculated the prevalence ratio to compare LF prevalence between groups with or without certain characteristics.

This study was approved by the Research Ethics Committee, n° 01-431.

## Results

A total of 333 patients met the inclusion criterion during the monitoring period. The mean age of sample was 32.40 years (SD = 18.70). The characteristics of the total sample are shown in Table 2.

**Table 2:** Characteristics of the total sample.

*Prevalence*	
Adults	217 (65.2%)
Women	180 (54.1%)

*Cholesteatoma growth pattern (prevalence)*	
Anterior epitympanic	6 (1.8%)
Posterior epitympanic	112 (33.6%)
Posterior mesotympanic	110 (33.0%)
Two-route	48 (14.4%)
Undetermined	57 (17.1%)

**Figure 1:**
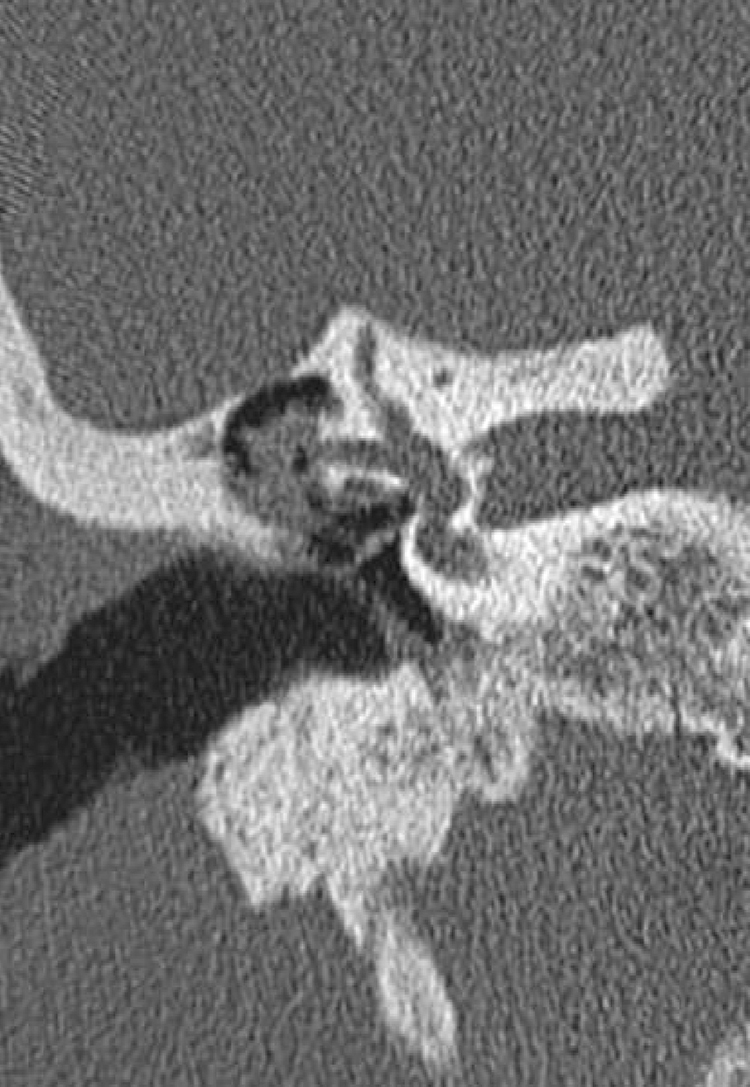
LF (lateral semicircular canal) in a CT scan.

LF was observed in only 9 (2.7%) patients. In all but one patient, the fistula was identified on CT scan and confirmed during surgery. Fig. 1 demonstrates a LF in CT scan. The mean time between CT scan and surgery was 10.75 months (SD = 6.98). One patient did not undergo surgery because of lung neoplasia and fistula was identified only on CT. All LF were in the lateral semicircular canal. The trans-operative classification of the fistula was Type II in seven patients and Type III in one. LF was not an incidental finding during surgery in any patients with acquired middle ear cholesteatoma.

The characteristics of patients with fistula are shown in Table 3.

**Table 3:** Characteristics of patients with labyrinthine fistula.

*Age (mean, standard deviation)*	44.44 (12.19)
*Adults*	9 (100%)
*Men (prevalence)*	5 (55.6%)
*Preoperative vertigo symptoms*	7 (77.8%)
*Preoperative sensorineural hearing loss*	3 (33.3%)

*Cholesteatoma growth pattern (prevalence)*	
Anterior epitympanic	0 (0%)
Posterior epitympanic	6 (66.7%)
Posterior mesotympanic	0 (0%)
Two-route	2 (22.2%)
Undetermined	1 (11.1%)
Abnormal contralateral ear	9 (100%)
Cholesteatoma	3 (33.3%)
Moderate or severe tympanic membrane retraction	6 (66.7%)

The mean age of patients with LF was 44.44 years (SD = 12.19); while in patients without fistula the mean age was 32.07 (SD = 18.75), *p* = 0.05. The mean duration of symptoms in LF patients was 19.71 months (SD = 14.61) and in patients without LF, 12.83 months (SD = 13.07), *p* = 0.14.

**Figure 2:**
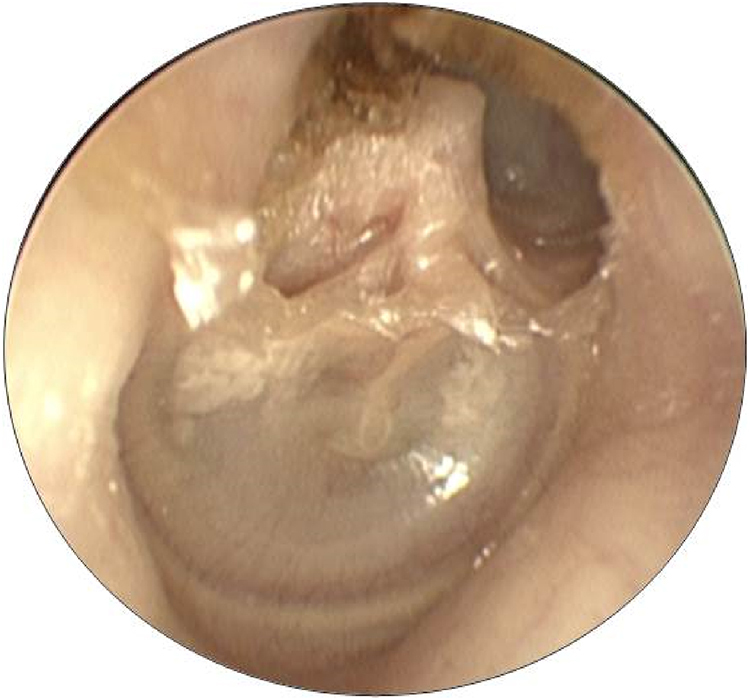
Posterior epitympanic cholesteatoma.

**Figure 3:**
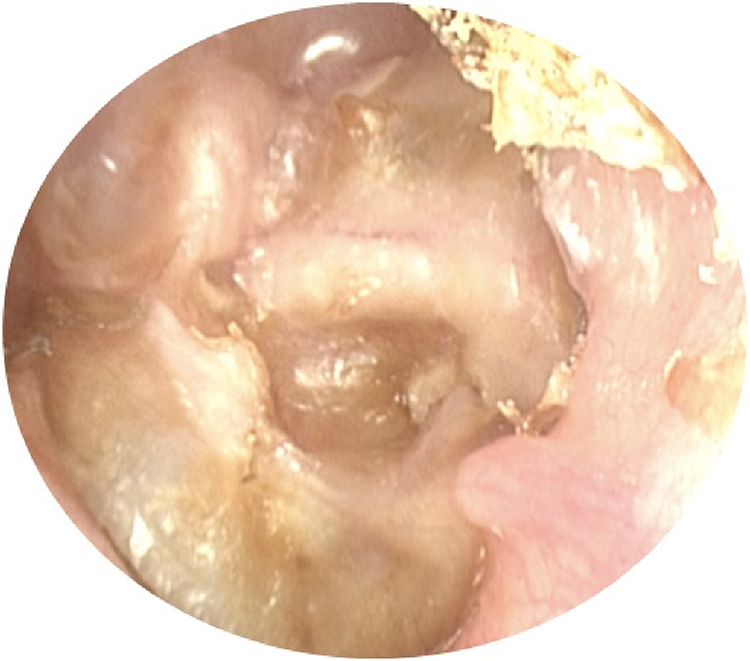
Two-route cholesteatoma.

The prevalence of LF in patients with cholesteatoma and vertigo complaint was 5.6% and in those without this symptom was 1.0% (*p* = 0.03). The prevalence ratio for LF between patients with and without vertigo was 2.1. The prevalence of LF in patients with PEC and two-route cholesteatomas was 5.0%, and in the remaining cholesteatoma, growth patterns was 0.6% (*p* = 0.16). Figure 2, Figure 3 demonstrate, respectively, a PEC and a two-route cholesteatoma, which were the cause of the LF in these patients. The prevalence ratio for LF between cholesteatomas that implicated the attic (PEC and two-route) and those that did not involve this site (PMC, anterior epitympanic, and undetermined) was 8.33.

We had 9 patients with LF: five without SHL before surgery, three with SHL previous to the surgery and one which the surgery was not performed due to adverse clinical condition. Considering the 5 patients without SHL before surgery (Type II fistula submitted to complete removal of cholesteatoma matrix), 4 (80.0%) remained with the same BC thresholds and 1 (20.0%) progressed to profound hearing loss. Considering the 3 patients with SHL before surgery (two with Type II and one with Type III fistula), 1 (33.33%) sustained the same hearing impairment and 1 (33.33%) showed improvement of the BC thresholds’ PTA. One patient (33.3%) with previous SHL, which had a Type III fistula and the technique with retention of matrix, was performed, progressed to profound hearing loss after surgery.

## Discussion

Among 333 patients with cholesteatoma, the prevalence of LF was 2.7%. The reported incidence of fistula during surgery for chronic otitis media together with cholesteatoma in different geographical areas and countries ranges between 2.9% and 12.5%.^10^, ^11^, ^12^ The relatively lower prevalence observed in our study is possibly due to inclusion of only patients without previous ear surgery. Therefore, all patients in our study had fistulas located in the lateral semicircular canal. Grewal et al.^11^ reported that the location of the fistula was the lateral semicircular canal in 96%, and Faramarzi^12^ reported the same location in 95.8% patients. Most studies show that the incidence of isolated lateral canal fistulas is about 80% and ranges from 57% to 91%.^13^, ^14^

CT is important in the pre-operative identification of LF. In previous studies, CT showed 50% sensitivity in the diagnosis of LF. However, more recent studies have reported sensitivity between 85% and 100%, owing to high resolution CT and thinner cuts (0.5 mm slices).^13^, ^14^, ^15^ CT is also able to predict the presence of a membranous fistula versus a bone fistula with sensitivity of 66% and specificity of 71%.^15^ In our study population, high resolution CT identified LF in all patients, which was confirmed at surgery. The high sensitivity and specificity in our study can be explained by the selection criteria, since we only included patients with LF Type II or Type III with evident bone erosion. Currently, CT scan is mandatory for all patients prior to cholesteatoma surgery for the analyses of anatomic landmarks, disease extension, and mastoid pneumatization, and subsequently, selection of the optimal surgical technique by case. The successful role of CT in pre-operative prediction of LF also facilitates cholesteatoma evaluation.

Only 33% of our patients had SHL before surgery, suggestive of the need for careful surgical management. Many techniques are available for patients with fistula.^16^ It is possible to use a closed or open technique for mastoidectomy. We prefer the latter, since it allows better exposure and manipulation of the underlying pathology. It is possible to leave the matrix of the cholesteatoma over the fistula or remove it, with a higher risk of cerebrospinal fluid (CSF) leak. A major concern is the capacity of the matrix to sustain bone erosion and cause delayed SHL. However, this can be less aggressive than a CSF leak followed by closure with temporalis fascia. If the matrix is removed, the fistula can be closed with (1) temporalis fascia, (2) temporalis fascia and fibrin glue, or (3) temporalis fascia and bone pate.

Published data on hearing loss are conflicting;^17^, ^18^, ^19^, ^20^, ^21^, ^22^ thus, the applicability of one technique in all LF cases is questionable. Clinical management should be based on the surgeon's ability and size of the fistula in each case. It may be necessary to use intra- and post-operative corticosteroids and antibiotics even in cases with hearing preservation, although the literature lacks consistent evidence of benefit.^21^, ^22^ However, the choice of a less aggressive technique with retention of the cholesteatoma matrix over the LF can also result in profound SHL, as in one of our patients.

The hearing result after surgery is even more important, considering that all patients with LF in the involved ear also had significant alterations in the contralateral ear, with cholesteatoma in 33.3%.

Our results also demonstrated that patients with LF were older than those without, probably because the erosion of the endochondral bone depends on cholesteatoma progression over the time. However, in this study, we failed to show that patients with LF have longer duration of symptoms. This finding could be due to the small sample of patients with LF in our study. On the other hand, data regarding the duration of disease have poor reliability,^23^ since the duration depends on patient's memory, previous symptoms of ear pain, otorrhea, or hypoacusis associated with a history of recurrent otitis media or chronic secretory otitis, which can be easily confused with the symptoms of cholesteatoma.

Interestingly, most patients with LF had a cholesteatoma growth pattern that involved the pars flaccida; and all but one had a PEC or a two-route cholesteatoma. Thus, patients with these growth patterns have a higher risk of LF in the lateral semicircular canal. PEC grows in the aditus direction, progressing to the lateral semicircular canal area earlier than cholesteatomas that arise in the pars tensa. Vertigo and SHL can consequently be more prevalent in these patients. Our previous study showed greater conductive hearing loss in patients with PMC, since it grows over the incus, the erosion of which has greater repercussions for hearing thresholds.^24^

## Conclusion

Labyrinthine fistula must be ruled out prior to ear surgery in adult patients with vertigo, particularly posterior epitympanic or two-route cholesteatoma. CT is a good diagnostic modality for lateral semicircular canal fistula. Regardless of the technique used to seal the fistula, adequate clinical management is needed, since sensorineural hearing loss can occur even in previously unaffected patients and significant alterations in the contralateral ear are not uncommon.

## Conflicts of interest

The authors declare no conflicts of interest.
